# Exploring Communication Practices in Italian Physiotherapy: Knowledge and Use of Effective Communication Strategies—A National Descriptive Study

**DOI:** 10.3390/healthcare11162247

**Published:** 2023-08-10

**Authors:** Mohammad Al-Wardat, Mohammad Etoom, Francesco Lena, Leonardo Pellicciari, Francesco D’Amone, Oyéné Kossi, Fabrizio Brindisino, Auwal Abdullahi

**Affiliations:** 1 Department of Rehabilitation Sciences, Faculty of Applied Medical Sciences, Jordan University of Science and Technology, Irbid 22110, Jordan; 2 Department of Physical Therapy, Aqaba University of Technology, Aqaba 77110, Jordan; metoom@aut.edu.jo; 3 IRCCS INM Neuromed, Department of Neurology, 86077 Pozzilli, Italy; 4 IRCCS Istituto delle Scienze Neurologiche di Bologna, 40139 Bologna, Italy; leonardo.pellicciari@ausl.bologna.it; 5 Department of Medicine and Health Science, “Vincenzo Tiberio”, University of Molise, 86100 Campobasso, Italy; damo1993@libero.it (F.D.); fabrizio.brindisino@unimol.it (F.B.); 6 ENATSE, National School of Public Health and Epidemiology, University of Parakou, Parakou 03 BP 10, Benin; oyene.kossi@uhasselt.be; 7 Unit of Neurology and NeuroRehabilitation, University Hospital of Parakou, Parakou 01 BP 02, Benin; 8 Department of Physiotherapy, Bayero University, Kano 700271, Nigeria

**Keywords:** communication, verbal language, non-verbal language, clinician–patient interaction, therapeutic adherence, physiotherapy

## Abstract

This study aimed to investigate the knowledge and use of effective communication strategies among Italian physiotherapists. We utilized a questionnaire consisting of 19 questions to collect data on the knowledge and use of effective communication strategies among Italian physiotherapists. The results revealed that only 35.8% of the respondents reported being aware of communication strategies related to physiotherapy, with their first exposure occurring during their three-year degree. Despite the majority of respondents agreeing that communication is an effective strategy for improving patient adherence, only about half reported making moderate use of open-ended questions and metaphors during treatment sessions. Furthermore, more than half of the respondents reported being unaware of Motivational Interviewing. The results of this study found that there is a consensus among Italian physiotherapists about the importance of effective communication in clinical practice, though the knowledge and application of some communication strategies remain limited. These findings suggest that there is room for improvement in the training and education of physiotherapists in Italy, with a need for greater emphasis on communication strategies in the university educational curriculum, starting from the bachelor’s degree.

## 1. Introduction

Several studies and reviews established that using appropriate therapeutic communication strategies is essential for enhancing therapeutic adherence and patient satisfaction and is a fundamental requirement for rehabilitation based on the biopsychosocial model [1,2,3]. Notably, improved therapeutic adherence has been linked to reduced pain and disability and increased patient satisfaction [4]. Furthermore, the first axiom of communication asserts that communication is inevitable, underscoring the vital importance of effective communication skills for physiotherapists, as this represents their primary direct interaction with patients [5]. Within the medical field, two recognized forms of communication (i.e., verbal and non-verbal communication) have distinct impacts on message reception. Additionally, a third form of communication (i.e., paraverbal communication, incorporating tone, timbre, and volume of voice) is recognized in the psychological field, which is considered part of non-verbal communication in the medical field. In this regard, Mehrabian’s “7% 38% 55% rule” (1981) is widely known but currently widely criticized [6]; this rule suggests that verbal, non-verbal, and paraverbal communication impact message decoding by 55%, 38%, and 7%, respectively.

Verbal communication refers to the meaning conveyed by words to transmit a message. In contrast, non-verbal communication includes gestures, posture, eye contact, attention, and tone and voice timbre, which also play a role in message transmission [7]. The SOLER acronym (Sits squarely, Open posture, Lean towards the other, Eye contact, Relax) was introduced by psychologist Gerard Egan in the 1970s to teach non-verbal language. Still, it has been criticized and replaced by SURETY (i.e., Sit at an angle to the client, uncross legs and arms, Relax, Eye contact, Touch, Your intuition) by Stickley [8,9]. Both verbal and non-verbal communication are encompassed within the context of the patient–physiotherapist relationship, which has been shown to influence therapeutic outcomes positively or negatively in numerous studies and questionnaires [10,11]. At the neurobiological level, contextual factors can stimulate specific neurotransmitters, such as cholecystokinin, opioids, endocannabinoids, vasopressin, and dopamine, which are responsible for significant clinical effects [10,11].

Indeed, previous studies indicated that communication skills training leads to a noteworthy improvement in medication adherence [12]. Specifically, a physician receiving communication training is 2.16 times more likely to engage the patient in adequate medication adherence than those without such training [2]. However, assessing a clinician’s communication abilities can be challenging since communication is a skill that is difficult to measure objectively. In the literature, some studies present evaluation scales for assessing communication skills. For example, Arthur (1999) proposed the Simulated Client Interview Rating Scale (SCIRS) to evaluate the communication skills of nursing students in Australia [13]. In another study conducted on a population of Irish physiotherapists in 2019, the reliability and validity of the Communication Evaluation in Rehabilitation Tools (CERT) scale were evaluated to assess communication skills [14].

Various studies have shown that clinicians tend to interrupt their patients within the first twenty seconds of their initial exposition of the problem that brought them to the clinic [14,15]. Beckman and Frankel (1984) found that interruptions occurred between 5 and 50 s after the clinician initially requested information [16]. However, patients who can complete the presentation of their problem generally do not take more than 60 s and certainly not more than 150 s. This tendency towards interruption may compromise the collection of anamnestic data and result in the loss of important information [16]. In situations where interruption is deemed necessary, healthcare professionals can use communication skills to remain in a patient-centered communication context. Ideally, patients should not be interrupted during the initial stages of an interview, especially when responding to open-ended questions [17].

Motivational Interviewing (MI) is a communication strategy extensively studied and supported by the literature [18]. It is a collaborative communication style that aims to reinforce the personal motivation of patients towards a specific goal, leading them to commit to a change without forcing or imposing beliefs [19]. Furthermore, it is conducted empathetically, supporting motivation and consolidating commitment [18]. A meta-analysis demonstrated that MI can improve short-term adherence to treatment for chronic pain and can increase adherence to exercise, self-efficacy, and the long-term maintenance of an active lifestyle [18]. A study by Oosterhof revealed that in chronic patients, satisfaction with the treatment is influenced by how the pain is explained to them [19]. Physiotherapists who are trained in MI techniques can effectively apply various principles such as collaboration, strengthening self-efficacy, and addressing barriers to their practice, thereby helping to increase adherence to physiotherapy treatment [20]. Additionally, MI can foster intrinsic motivation and empower clients to make positive changes in their health behaviors [20,21]

Overall, using metaphors and effective communication skills training is crucial for clinicians [19]. This highlights the importance of knowledge and the use of therapeutic communication for physiotherapists, as it represents the first therapeutic interaction with the patient. Indeed, the communication between a physiotherapist and a patient plays a crucial role in improving rehabilitation outcomes. Specifically, effective communication enhances the therapeutic relationship and promotes collaboration, understanding, and trust between the physiotherapist and the patient [22]. Thus, this study investigates the level of understanding and implementation of effective communication strategies among Italian physiotherapists in their daily clinical practice. In addition, the study explores how communication is integrated into clinical practice and examines the extent to which communication strategies are understood and utilized by physiotherapists in Italy.

## 2. Materials and Methods

### 2.1. Study Design

The present study is a cross-sectional web-based survey conducted nationwide that adheres to the CHERRIES and STROBE guidelines for reporting online surveys and observational studies [23,24]. The Institutional Review Board (IRB) at the University of Molise approved all the study procedures (approval number 20/2021).

### 2.2. Participants and Methods of Administration of the Survey

All participants in the study were Italian physiotherapists who voluntarily and anonymously participated in the survey without receiving any compensation. The inclusion criteria were as follows: (1) Licensed and registered physiotherapists in Italy. (2) Should have a minimum of one year of professional experience in the field of physiotherapy. (3) Physiotherapists of all specialties and practice settings, including hospitals, private clinics, rehabilitation centers, and community-based settings, are eligible to participate. (4) Participants must be proficient in the Italian language to ensure effective communication during the study. We excluded the following: (1) Students or individuals who are not licensed and registered physiotherapists. (2) Participants with less than one year of professional experience in physiotherapy. (3) Physiotherapists who do not practice in Italy or are not currently residing in the country. The online survey was conducted using “SurveyMonkey” software, and the questionnaire was distributed through various physiotherapy groups on social media platforms such as Facebook, Instagram, and WhatsApp.

### 2.3. Development of the Questionnaire

A preliminary literature search was conducted on communication in healthcare, revealing the challenge of standardizing an objective evaluation method. No surveys with the same population and purpose as this study were found. A group of experienced physiotherapists and clinicians from the University of Molise compiled a preliminary list of 19 questions in the Italian language, which included sociodemographic and cognitive/application questions. These questions were developed through an intensive literature review and a series of discussions among the research team members and experienced communication clinicians to ensure the validity of their content. Seven sociodemographic and twelve cognitive/application questions were then selected and standardized by a psychologist experienced in communication and questionnaire administration. The questionnaire was reviewed by seven physiotherapists not involved in the study to evaluate question clarity and timing. The finalized questionnaire in the Italian language was uploaded to the SurveyMonkey platform for web-based administration. The sample size was calculated using the number of physiotherapists in the latest Italian Association of Physiotherapy census (64,688). This resulted in a target of 382 respondents for a 95% confidence interval and a 5% error rate. Before accessing the questionnaire, participants were informed of the study’s purpose, estimated time commitment (approximately 3 min), and total anonymity of the collected data. A pilot test was conducted involving 10 people (PTs and non-PTs) to assess the logic and clarity of the survey. Feedback from the pilot survey was incorporated before the final version was confirmed. The internal consistency was assessed using Cronbach’s alpha (0.72), which indicated acceptable overall internal consistency. The translated questionnaire (English version) is available in its entirety in the Appendix A.

### 2.4. Data Collection and Analysis

On 26 May 2022, the questionnaire was uploaded and made available to participants through an invitation link on the SurveyMonkey platform, which automatically imported respondents’ answers into an Excel document upon completion. At the end of October 2022, the data collection ended. A descriptive analysis was initially performed to describe the sample characteristics, identifying each variable’s frequency, percentage, and 95% confidence interval (95% CI).

## 3. Results

### 3.1. Sociodemographic Characteristics of the Respondents

**Scheme 1:**
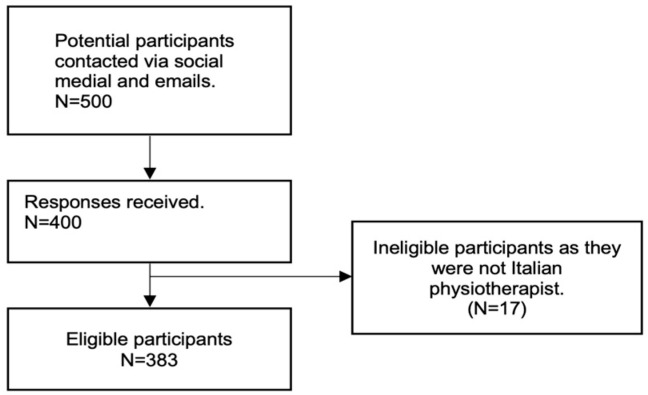
The participant recruitment process.

Out of the 400 responses received, 383 participants were included based on the study’s inclusion and exclusion criteria (see Scheme 1), while 17 responses were excluded according to these criteria. The age distribution of the respondents was as follows: 182 (47.5%) were between 26 and 34 years old, 89 (23.2%) were between 35 and 50 years old, 64 (16.7%) were between 21 and 25 years old, and 48 (12.5%) were over 50 years old. In terms of gender, 189 (49.3%) respondents were female and 196 (50.7%) were male. Regarding the geographical distribution of the respondents, the majority, 208 (54.3%), worked in the South (Abruzzo, Apulia, Basilicata, Calabria, Campania, and Molise) and Islands region (Sicily and Sardinia) of Italy, while 95 (24.8%) worked in Northern Italy (Milan, Turin, Venice, Bologna, Verona, Pisa, Genoa, Florence, Padua, and Trieste) and 80 (20.9%) worked in Central Italy (Rome, Siena and Umbria). In terms of qualifications, 64.5% of the respondents held a three-year degree, 27.4% held a first or second-level university master’s degree, and 8.1% had a specialist degree.

**Figure 1:**
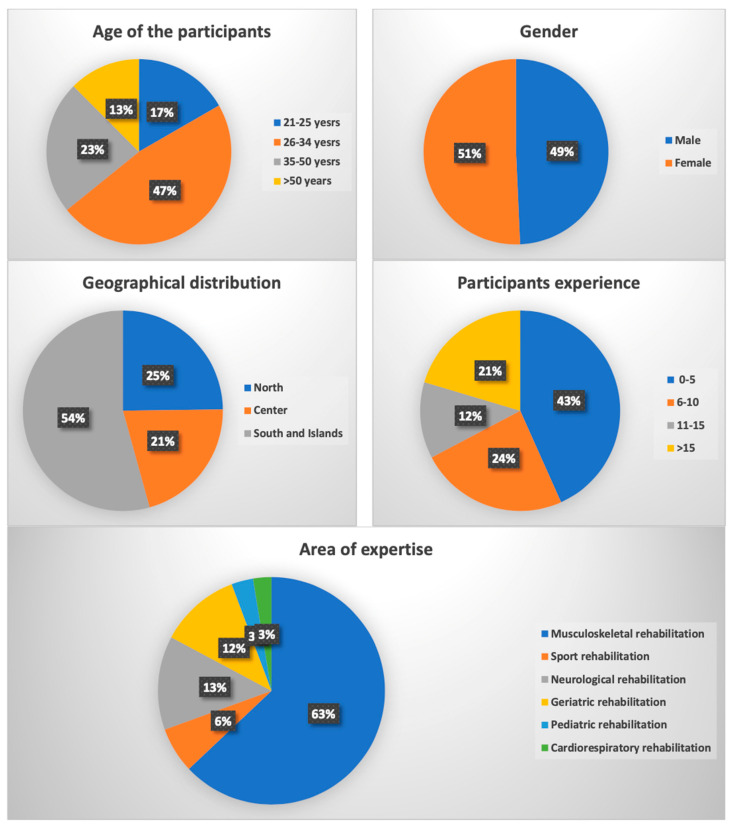
Characteristics of the participants (% = percentage).

In terms of work experience, 43.3% of the respondents reported having 1–5 years of work experience, 24% reported having 6–10 years, 12.3% reported having 11–15 years, and 20.4% reported having more than 15 years of experience. The majority of the respondents, 65.3%, identified as freelancers, while 34.7% identified as employees. The most reported field of work among the respondents was musculoskeletal rehabilitation, with 241 (62.9%) respondents indicating this as their area of expertise. This was followed by neurological rehabilitation, geriatric rehabilitation, sports rehabilitation, pediatric rehabilitation, and cardiorespiratory rehabilitation fields. Figure 1 provides further details on the characteristics of the participants.

### 3.2. Results of the Questions in the Understanding and Implementation of Effective Communication Strategies

In the survey, participants were asked about their first exposure to communication pertaining to the profession of physiotherapist. Results revealed that the majority of participants (35.8%, *n* = 137) became aware of this communication during their three-year degree. A significant proportion (20.9%, *n* = 80) first encountered it during a university master’s degree. A smaller percentage (14.6%, *n* = 56) reported learning about it through a private course, while an equal percentage (14.9%, *n* = 57) provided varied responses. Interestingly, a portion of participants (8.6%, *n* = 33) reported never having heard of such communication. A minority (2.9%, *n* = 11) first encountered it during a university course, and a slightly smaller minority (2.3%, *n* = 9) became aware of it during a master’s degree program.

In relation to the question about non-verbal language, the majority of participants (72.1%, *n* = 276) provided the correct response that using metaphors to explain concepts to the patient is not an example of non-verbal language. However, a notable portion (10.7%, *n* = 41) incorrectly identified “physiotherapist gestures” as not being an example of non-verbal language, while a smaller percentage (6%, *n* = 23) believed that “physiotherapist eye movements” do not represent non-verbal language. Furthermore, 8.4% (*n* = 32) of participants identified the “tone and timbre of voice used by the physiotherapist” as not being an example of non-verbal language, and 2.9% (*n* = 11) displayed unfamiliarity with the distinction between verbal and non-verbal language.

During the patient interview, a significant majority of respondents (75.7%, *n* = 290) reported paying a lot of attention to their word choices and usage. A smaller percentage (22.7%, *n* = 87) indicated moderate attention, while only 1% (*n* = 4) reported giving little attention to this aspect. Moreover, when queried about the potential harm caused by words, a substantial portion (86.7%, *n* = 332) strongly agreed, 10.7% (*n* = 41) moderately agreed, and a minority (1.3%, *n* = 5) either slightly or strongly disagreed.

In terms of the effectiveness of therapeutic communication, a significant majority (88.5%, *n* = 339) considered it to be a highly effective strategy for improving patient adherence. A smaller proportion (10.2%, *n* = 39) rated it as moderately effective, while only 1% (*n* = 4) perceived it as not very effective. Negligibly, only 0.3% (*n* = 1) regarded it as not at all effective.

When inquiring about the duration of time spent with the patient to ensure their understanding of discussed concepts during the session, various response patterns emerged. Approximately 14.9% (*n* = 57) of participants reported allocating around 2 min for this purpose. A larger proportion, 30.5% (*n* = 117), dedicated 3 to 5 min, while 29% (*n* = 111) extended the duration to 6 to 10 min. A notable percentage, 19.6% (*n* = 75), invested more than 10 min for this task. However, a small percentage (6%, *n* = 23) did not provide a response.

Regarding the strategies employed to enhance patient comprehension during sessions, various approaches were reported. The majority of participants (58.7%, *n* = 225) utilized verbal communication, while a notable proportion (18.5%, *n* = 71) used visual aids such as images. Additionally, 10.7% (*n* = 41) utilized audiovisual material or online resources, 6.8% (*n* = 26) employed other strategies, and 3.1% (*n* = 12) utilized booklets. Interestingly, a small percentage (2.1%, *n* = 8) did not use any supporting materials. When queried about the duration of waiting before interrupting the patient during the anamnestic collection, responses varied. Approximately half of the participants (50.4%, *n* = 193) reported allowing the patient to speak until they naturally interrupted. Around 31.6% (*n* = 121) waited for about 2 to 3 min before intervening, while 15.9% (*n* = 61) waited for approximately 1 min. Only a small percentage (2.1%, *n* = 8) reported stopping the patient after just a few seconds. After identifying the patient type, the majority of respondents (93.7%, *n* = 359) reported using a communication register similar to that of the patient. In contrast, a minority (6.3%, *n* = 24) adhered to their own communicative register.

More than half of the participants (55.4%, *n* = 212) indicated that they had not heard of “MI”, while the remaining 44.6% (*n* = 171) reported being familiar with it. Regarding the use of open questions during anamnesis, the majority of respondents (54.6%, *n* = 209) reported using them moderately, while a significant portion (25.6%, *n* = 98) used them extensively. A smaller percentage (18%, *n* = 69) reported using open questions to a lesser extent, and only 1.8% (*n* = 7) reported not using them at all. Concerning the use of metaphors during treatment sessions, a majority of respondents (52%, *n* = 199) reported using them moderately, while 30.3% (*n* = 116) reported using metaphors frequently. A smaller percentage (13.8%, *n* = 53) reported using metaphors sparingly, and 3.9% (*n* = 15) reported not using them at all. The respondents’ answers to the understanding and implementation of effective communication strategies questions in the questionnaire are summarized in Table 1.

## 4. Discussion

This study presents the first investigation in Italy on the knowledge and utilization of fundamental aspects of communication during clinical practice among Italian physiotherapists. The findings reveal that slightly over 30% of the surveyed physiotherapists first learned about communication in their profession during their three-year degree—a relatively low percentage considering the potential benefits of correct therapeutic communication, including enhanced therapeutic adherence and patient satisfaction, both of which are integral to rehabilitation grounded in the biopsychosocial model [1,2,3]. The literature further suggests that physiotherapists with communication training are better equipped to support patients than those without such training [25]. Furthermore, the communication skills of the physiotherapist are recognized as an influential factor in the therapist–patient interaction, which has been shown to impact treatment outcomes [26].

The present study indicates that individuals with a bachelor’s degree in physiotherapy were more commonly found in the younger age bracket (21–25 years) and had limited work experience (0–5 years). These findings suggest that the educational programs for three-year physiotherapy degrees in Italian universities may be evolving to emphasize topics such as therapeutic communication. Additionally, the majority of participants (72.1%) demonstrated an understanding of the difference between verbal and non-verbal language, with older physiotherapists (≥50 years) and those with more experience (>15 years) exhibiting a lower level of accuracy in their responses. These observations support the notion that younger physiotherapists with less experience may be more aware of the importance of communication-related topics.

The results of the survey indicate that the interviewed physiotherapists are highly attentive to the language used during therapy sessions, recognizing the potential harm that words can cause to the patient and the resulting nocebo effect. This finding is consistent with previous research that suggests negative verbal suggestions can induce anticipatory anxiety and trigger the activation of cholecystokinin (CCK), ultimately facilitating pain transmission [27]. Furthermore, a previous study has shown that negative or pain-related verbal stimuli can activate specific brain regions [28]. These findings align with the current understanding of the importance of using positive and supportive language during therapeutic interactions, particularly in pain management [29,30].

Most respondents (88.5%) identified the use of effective therapeutic communication as a highly effective strategy for improving patient therapeutic adherence. This finding is consistent with the literature, which indicates that effective communication can significantly enhance a patient’s likelihood of adhering to treatment. One meta-analysis in particular has demonstrated that patients are 2.16 times more likely to adhere to treatment when the healthcare professional communicates effectively [2].

The results of this survey suggest that Italian physiotherapists allocate a significant amount of time to communicating with their patients to ensure the comprehension of the information imparted during treatment sessions. Most respondents (59.5%) reported spending between 3 to 10 min on this aspect. Expressly, 30.5% of physiotherapists indicated spending between 3 and 5 min, while 29% reported spending between 6 and 10 min. Interestingly, only a small minority (6%) of respondents reported spending no time on this aspect. Notably, physiotherapists over 50 years were likelier to allocate more than 10 min to this activity. These findings are consistent with the literature, suggesting that patients with unfavorable treatment outcomes do not arrive at a shared understanding of their pain with the healthcare professional treating them, particularly in chronic pain [19].

Regarding the methods used to facilitate patient comprehension of the concepts presented during the session, most Italian physiotherapists (58.7%) reported relying on verbal communication, followed by the use of visual aids such as images (18.5%), audiovisual material or online resources (10.7%), and reading materials such as brochures (3.1%). However, this finding appears to diverge from the literature, which suggests that using an information booklet can reduce disability in patients with subacute and chronic lower back pain [31]. Further research would be valuable in determining which form of support impacts patient understanding the most.

Interrupting patients during the anamnestic collection can be a delicate matter. Clinicians need to balance allowing the patient to express themselves fully and efficiently and gathering relevant information [16,32]. The use of the three “Es” (excuse, empathize, explain) can be a useful strategy for clinicians to interrupt patients in a respectful and empathetic way [17]. It is also worth noting that some patients may be more talkative than others, and clinicians should adapt their approach accordingly. Overall, finding the right balance between allowing the patient to speak and interrupting them, when necessary, can improve the quality of the anamnestic collection and ultimately lead to better patient outcomes [17].

Adapting to the communicative register of the patient is an essential aspect of therapeutic communication, as it helps to establish a good rapport with the patient and facilitates the exchange of information. Interestingly, physiotherapists who have been practicing for more than 15 years showed a lower tendency to adapt to the communicative register of the patient. This may be because they have developed their communication style over the years and may find it difficult to change. However, all physiotherapists need to be aware of the importance of adapting to the patient’s communicative register, as it can significantly impact the therapeutic relationship’s success [30].

It is important to mention that effective communication in physiotherapy practice should involve the use of both verbal and non-verbal feedback. Verbal (words, tone, and content of a person’s response) and non-verbal (facial expressions, body language, gestures, and other non-spoken cues) feedback have a significant impact on communication [33]. Specifically, verbal feedback provides clear information for understanding, while non-verbal feedback enhances emotional cues and context to increase comprehension. Verbal feedback enables physiotherapists to provide clear and detailed information to patients, ensuring that instructions and treatment plans are well understood [33,34]. Non-verbal feedback, through facial expressions, body language, and other cues, enhances the emotional connection between physiotherapists and patients, conveying empathy and fostering a sense of understanding and support [33,34]. By combining both types of feedback, physiotherapists can establish a strong rapport and optimize the therapeutic relationship with their patients [35].

MI is a counseling approach developed to help people change their behavior by resolving ambivalence, increasing intrinsic motivation, and creating a sense of empowerment [20,21]. It is often used in healthcare settings to address behavioral changes related to health issues such as chronic pain, addiction, and obesity [20,21]. However, the fact that more than half of the Italian physiotherapists interviewed were unaware of this strategy highlights the need for further education and training in communication skills and evidence-based interventions [36,37]. According to these results, additional education and training programs focused on communication skills and evidence-based interventions could be beneficial for Italian physiotherapists. These programs could address the lack of awareness of the MI strategy among many practitioners and provide them with the necessary knowledge and tools to integrate it into their practice. By investing in professional development, healthcare systems can promote the delivery of high-quality care and improve patient outcomes.

Using open-ended questions during the anamnestic collection can help to elicit more detailed information from patients and provide a more comprehensive understanding of their condition. Therefore, it is encouraging that most interviewees reported using open-ended questions moderately or frequently. However, physiotherapists from all regions need to recognize the value of open-ended questions and strive to incorporate them more into their patient interactions [38,39,40].

It is important to note that metaphors can be a powerful tool to facilitate communication and understanding between the clinician and the patient. It allows complex concepts to be explained and understandably. Furthermore, studies have shown that metaphors can improve patient satisfaction, the understanding of the disease and treatment, and treatment adherence [41]. Therefore, it is recommended that Italian physiotherapists receive more training on the use of metaphors in their clinical practice.

It would be desirable to increase the use of metaphors since the literature suggests that they can help patients to better understand and restructure beliefs, including those related to pain [41,42]. Moreover, a patient’s complete comprehension of pain is a crucial factor in the treatment of chronic pain and in ensuring high-quality clinician–patient interactions. This can be facilitated by using simple words and metaphors [19].

The study has several strengths, including the efficient and effective methodological approach that allowed for a large sample of healthcare professionals to be surveyed quickly. Similar studies have been conducted to examine the knowledge of Italian physiotherapists on communication and other contextual factors [43]. Additionally, the gender ratio of the interviewees is evenly balanced, preventing gender biases. Future studies are recommended to consider the stratification of the sample by region to mitigate any potential biases and enhance the generalizability of findings.

However, it is important to acknowledge the limitations of this study. Most of the respondents (65.3%) work as freelancers and work with musculoskeletal therapy (62.9%), which may limit the generalizability of the findings to the entire population of physiotherapists. Additionally, over half of the interviewees (54.3%) were from Italy’s Southern and Island regions, which may compromise the sample’s representativeness. The assessment tool used in this study was primarily focused on the level of understanding and implementation of effective communication strategies. However, it is important to note that the survey answers were not related to a Likert scale, which made it challenging to obtain quantitative scores. Thus, it is important to note that this study is primarily descriptive in nature. While it provides valuable insights into communication practices in the context of Italian physiotherapy, further research is highly recommended to delve into potential associations between responders’ characteristics and communication practices. To achieve this, future studies should employ appropriate statistical analyses, such as regression analysis. By exploring these associations, a more comprehensive understanding of the factors influencing communication practices can be gained, paving the way for the development of targeted interventions and improved patient outcomes. Despite the internal consistency of the survey being acceptable with a value of 0.72, we acknowledge that one limitation of our study is that the survey was administered in the Italian language. This may have implications for the generalizability of the findings to populations who are not fluent in Italian. In future studies, we highly recommend employing validated outcome measures and considering the use of multiple languages to enhance the applicability of the research findings across diverse populations. Lastly, it is important to acknowledge that this study primarily relied on subjective assessments obtained through a web-based questionnaire. While valuable insights were gathered through this approach, it is recommended that future studies incorporate objective assessment tests as well. By combining subjective and objective measures, a more comprehensive understanding of communication practices in Italian physiotherapy can be achieved. Objective assessment tests can provide additional quantitative data and further enhance the validity and reliability of the findings.

## 5. Conclusions

This study represents the first attempt to assess the use of effective communication strategies in the clinical practice of Italian physiotherapists. Unfortunately, while the findings indicate that Italian physiotherapists are mindful of communication aspects during clinical practice, the study also revealed that only slightly over 3 out of 10 physiotherapists had heard of communication-related topics during their three-year degree. This is a concerning figure as it is a relatively simple topic to cover and can immediately impact daily clinical practice. Therefore, improving the training provided by Italian universities in this regard is essential.

## Figures and Tables

**Table 1:** The respondents’ answers to the understanding and implementation of effective communication strategies questions (*n* = frequency, % = percentage, CI = 95% confidence interval).

Questions	*n* (%)	95% CI
In which context did you hear about communication related to the profession of physiotherapist “for the first time”?		
Private course	56 (14.6%)	11.1–18.2
University course	11 (2.9%)	1.2–4.5
Master’s degree	9 (2.3%)	0.8–3.9
Three-year degree	137 (35.8%)	31–40.6
University master’s	80 (20.9%)	16.8–25
I have never heard of it	33 (8.6%)	5.8–11.4
Other	57 (14.9%)	11.3–18.4
Among the following, which “non” represents an example of “non-verbal language”?		
Gestures of the physiotherapist.	41 (10.7%)	7.6–13.8
Physiotherapist eye movements.	23 (6%)	3.6–8.6
Using metaphors to explain concept to the patient.	276 (72.1%)	67.6–76.6
Tone and timbre of the physiotherapist’s voice.	32 (8.4%)	5.6–11.1
I don’t know the difference	11 (2.9%)	1.2–4.5
During the interview with the patient.		
I don’t pay attention to the words I use and how I use them	2 (0.5%)	−0.2–1.2
I pay little attention to the words I use and how I use them.	4 (1%)	0–2.1
I pay moderate attention to the words I use and how I use them	87 (22.7%)	18.5–26.9
I pay “a lot” of attention to the words I use and how I use them.	290 (75.7%)	71.4–80
In relation to the statement “Words can harm the patient,” please select the option that best aligns with your agreement:		
Not at all	5 (1.3%)	0.2–2.4
Slightly	5 (1.3%)	0.2–2.4
Moderately	41 (10.7%)	7.6–13.8
Strongly	352 (86.7%)	83.3–90.1
The use of effective therapeutic communication represents.		
A strategy “not at all” effective in increasing the patient’s therapeutic adherence.	1 (0.3%)	−0.2–0.8
An “ineffective” strategy in increasing the patient’s therapeutic adherence	4 (1%)	0–2.1
A “moderately” effective strategy in increasing the patient’s adherence.	39 (10.2%)	7.2–13.2
A “very” effective strategy to increasing the patient’s therapeutic adherence.	339 (88.5%)	85.3–91.7
How much time do you typically dedicate to ensuring that the patient has correctly understood the concepts conveyed during the session (e.g., summarizing aloud, inviting the patient to explain the learned concepts in their own words, watching summary videos together)?		
Approximately 2 min.	57 (14.9%)	11.3–18.4
3 to 5 min.	117 (30.5%)	25.9–35.2
6 to 10 min.	111 (29%)	24.4–33.5
More than 10 min.	75 (19.6%)	15.6–23.6
I do not engage in this practice.	23 (6%)	3.6–8.4
Which option among the following is utilized to facilitate the patient’s comprehension of the explanations provided during the session?		
Words.	225 (58.7%)	53.8–63.7
Images.	71 (18.5%)	14.6–22.4
Brochures to read.	12 (3.1%)	1.4–4.9
Audiovisual material or online resources.	41 (10.7%)	7.6–13.8
I don’t use any media.	8 (2.1%)	0.7–3.5
Other.	26 (6.8%)	4.3–9.3
During the collection of medical history, which approch do you typically follow regarding patient interruptions.		
Only a few seconds pass before interrupting the patient.	8 (2.1%)	0.7–3.5
Approximately 1 min passes before interrupting the patient.	61 (15.9%)	12.3–19.6
2 to 3 min pass before interrupting the patient.	121 (31.6%)	26.9–36.2
I let the patient talk until they stop on their own.	193 (50.4%)	45.4–55.4
Once the type of patient has been identified.		
I remain fixed on my communicative register.	24 (6.3%)	3.8–8.7
I use a communication register similar to that of the patient.	359 (93.7%)	91.3–96.2
Have you ever heard of “Motivational Interviewing”?		
No	212 (55.4%)	50.4–60.4
Yes	171 (44.6%)	39.7–49.6
During the medical history collection.		
I don’t use open-ended questions.	7 (1.8%)	0.5–3.2
I use “few” open questions.	69 (18%)	14.2–21.9
I use “moderately” open-ended questions.	209 (54.6%)	49.6–59.6
I use “many” open-ended questions.	98 (25.6%)	21.2–30
During the treatment session, please indicate your use of metaphors to help the patient better understand concepts:		
I “don’t” use metaphors to allow the patient to better grasp the concepts.	15 (3.9%)	2–5.9
I use “few” metaphors to allow the patient to better grasp the concepts.	53 (13.8%)	10.4–17.3
I use metaphors “moderately” to allow the patient to better grasp the concepts.	199 (52%)	47–57
I use “many” metaphors to allow the patient to better grasp the concepts.	116 (30.3%)	25.7–34.9

## Data Availability

The data that support the findings of this study are available from the corresponding author upon reasonable request.

## Appendix A

The complete questionnaire consists of 19 questions, covering both socio-demographic and effective communication strategies aspects. Questions Q1 to Q7 are of a socio-demographic nature, while questions Q8 to Q19 focus on the knowledge and application of effective communication strategies in clinical practice.

The questionnaire aims to evaluate the level of knowledge and application of effective communication strategies in the clinical practice of Italian physiotherapists.

Q1. How old are you?

- 21–25
- 26–34
- 35–50
- >50

Q2. Please select your gender from the following options:

- Female
- Male

Q3. Which specific geographic region serves as the primary location for your professional activities?

- North
- Center
- South and islands

Q4. What is the highest level of education you have attained?

- Three-year degree
- High diploma (1 year)
- Master’s degree

Q5. For how many years have you been actively practicing your profession?

- 0–5
- 6–10
- 11–15
- >15

Q6. Which of the following options best represents your preferred working environment?

- Employee
- Freelance

Q7. In which therapeutic area do you predominantly work?

- Musculoskeletal rehabilitation
- Sport rehabilitation
- Neurological rehabilitation
- Geriatric rehabilitation
- Pediatric rehabilitation
- Cardiorespiratory rehabilitation

Q8. In which context did you hear about communication related to the profession of physiotherapist “for the first time”?

- I never heard of it
- Three-year degree
- Master’s degree
- University Masters
- Private course university course
- Other

Q9. Among the following, which “non” represents an example of “non-verbal language”?

- Gestures of the physiotherapist.
- Physiotherapist eye movements.
- Using metaphors to explain concepts to the patient.
- Tone and timbre of the physiotherapist’s voice.
- I don’t know the difference between verbal and non-verbal language.

Q10. During the interview with the patient:

- I don’t pay attention to the words I use and how I use them.
- I pay little attention to the words I use and how I use them.
- I pay moderate attention to the words I use and how I use them.
- I pay “a lot” of attention to the words I use and how I use them.

Q11. In relation to the statement “Words can harm the patient,” please select the option that best aligns with your agreement:

- Not at all
- Slightly
- Moderately
- Strongly

Q12. The use of effective therapeutic communication represents:

- A strategy “not at all” effective in increasing the patient’s therapeutic adherence.
- An “ineffective” strategy in increasing the patient’s therapeutic adherence.
- A “moderately” effective strategy in increasing the patient’s therapeutic adherence.
- A “very” effective strategy in increasing the patient’s therapeutic adherence.

Q13. How much time do you typically dedicate to ensuring that the patient has correctly understood the concepts conveyed during the session (e.g., summarizing aloud, inviting the patient to explain the learned concepts in their own words, watching summary videos together)?

- I do not engage in this practice.
- Approximately 2 min.
- 3 to 5 min.
- 6 to 10 min.
- More than 10 min.

Q14. Which option among the following is utilized to facilitate the patient’s comprehension of the explanations provided during the session?

- I don’t use any media.
- Words.
- Images.
- Brochures to read.
- Audiovisual material or online resources.
- Other.

Q15. During the collection of medical history, which approach do you typically follow regarding patient interruptions?

- Only a few seconds pass before interrupting the patient.
- Approximately 1-min passes before interrupting the patient.
- 2 to 3 min pass before interrupting the patient.
- I let the patient talk until they stop on their own.

Q16. Once the type of patient has been identified:

- I use a communication register similar to that of the patient.
- I remain fixed on my communicative register.

Q17. Have you ever heard of “Motivational Interviewing”?

- Yes
- No

Q18. During the medical history collection:

- I don’t” use open-ended questions.
- I use “few” open questions.
- I use “moderately” open-ended questions.
- I use “many” open-ended questions.

Q19. During the treatment session, please indicate your use of metaphors to help the patient better understand concepts:

- “I don’t” use metaphors to allow the patient to better grasp the concepts.
- “I use few” metaphors to allow the patient to better grasp the concepts.
- “I use metaphors moderately” to allow the patient to better grasp the concepts.
- “I use many” metaphors to allow the patient to better grasp the concepts.
